# Development of a standardized and reproducible murine femoral distraction osteogenesis model

**DOI:** 10.1016/j.jot.2024.08.001

**Published:** 2024-10-04

**Authors:** Yuejun Lin, Zhaowei Jiang, Jiaming Yang, Ming Wang, Haixing Wang, Xiaoting Zhang, Xuan Lu, Shanshan Bai, Tongzhou Liang, Botai Li, Jie Shao, Lu Zhang, Dashuang Gao, Jiajun Chen, Sien Lin, Fan Yang, Gang Li

**Affiliations:** a Stem Cells and Regenerative Medicine Laboratory, Li Ka Shing Institute of Health Sciences, The Chinese University of Hong Kong, Prince of Wales Hospital, Shatin, Hong Kong, China; b Musculoskeletal Research Laboratory, Department of Orthopaedics & Traumatology, Faculty of Medicine, The Chinese University of Hong Kong, Prince of Wales Hospital, Shatin, Hong Kong, China; c The Brain Cognition and Brain Disease Institute, Shenzhen Institute of Advanced Technology, Chinese Academy of Sciences, Shenzhen, China; d The Guangdong Provincial Key Laboratory of Brain Connectome and Behavior, Shenzhen, Guangdong, China; e CAS Key Laboratory of Brain Connectome and Manipulation, Shenzhen, Guangdong, China; f Shenzhen-Hong Kong Institute of Brain Science-Shenzhen Fundamental Research Institutions, Shenzhen, Guangdong, China; g School of Medicine, Shenzhen Campus of Sun Yat-sen University, Shenzhen, China; h University of Chinese Academy of Sciences, Beijing, China

**Keywords:** Distraction osteogenesis, External fixators, Limb lengthening, Mouse model

## Abstract

**Objective:**

Distraction osteogenesis (DO) has been widely used to treat bone defects as its effectiveness in bone regeneration. Currently, distraction devices for establishing DO models are mainly developed for rats or large animals. However, a mouse DO model is in great need for in-depth mechanistic investigations using various transgenic mice. The current study reports the development of a reproducible murine DO model.

**Methods:**

A mini-titanium lengthener was designed and fabricated. The mini-lengthener was applied on the murine femur with four threaded pins using a designed clamp as the drilling and insertion guide. After transverse osteotomy using a Gigli saw, and after 5 days of latency, DO procedures started at 0.3 mm/day for 10 days, and the consolidation period was left for 28 days. The bone formation was monitored by radiography and histology. Potential effects on animal locomotion during DO were also measured by behavior tests.

**Results:**

Separated bone segments maintained good alignment during the entire DO phases. New bone formation was found as early as the end of the distraction phase. Active bone remodeling was found between the separated bone segments at late distraction and early consolidation phases. At the mature consolidation phase, bone remodeling was mainly observed in the contact cortical bone. Mice underwent DO procedure did not have significant impairment in their locomotion.

**Conclusion:**

We have successfully developed a murine femoral DO model, which may be used to study the biological processes of DO. We also developed the mini-lengthener and the guide clamp to ensure the standardization and reproducibility of the mouse DO model.

The translational potential of this article: Current study reports the development of a murine femoral DO model. A well-established murine DO model will facilitate further investigations of the biological mechanisms of DO in various transgenic and normal mice.

## Graphical abstract

**Figure:**
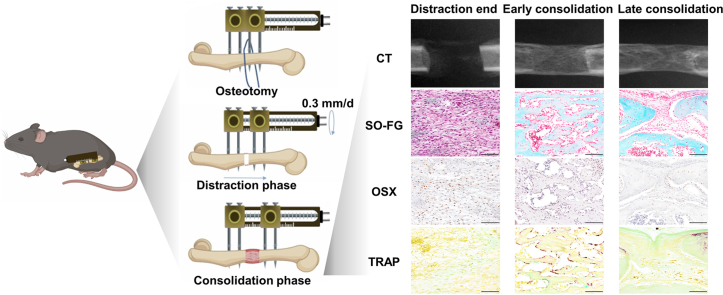

## 1 Introduction

Distraction osteogenesis (DO) was initially developed in the early twentieth century to treat extremely short limbs due to injury, disease, malformation, and fracture non-union [1,2]. DO has also been widely applied in the treatment of facial malformation, including Pierre–Robin sequence, Treacher Collins syndrome, and craniofacial microsomia [2], and in other difficult orthopaedic conditions such as limb deformities and segmental bone defects with outstanding outcomes [3]. During DO, tension forces promote endogenous bone formation in a well-controlled microenvironment, which is a new form of functional tissue engineering approach [4].

Despite the clinical advances using DO methods, the biological mechanisms of bone regeneration in DO still needs further exploration. Establishing a stable DO model is greatly needed for in-depth investigations of DO mechanisms. Current animal models of DO are mainly established using large animals, such as sheep [5], dogs [6], and rabbits [7]. Recent advances in transgenic mice provide possibilities for in-depth molecular mechanisms studies [8]. Hence, a mouse model of DO is of great need.

Fabricating a mini distraction device for mouse long bones with stable fixation is challenging. Helms et al. first reported an apparatus weighed 7 g and with an outer diameter of 20.5 mm for a mouse DO model in 1998 [9]. Simpson et al. [10] and Luyten et al. [11] also designed circular external fixation devices in mouse DO models with an outer diameter of about 18 mm and 14 mm, respectively. The outer diameter was about 10 times that of a mouse hind leg, which is inconvenient for mice to wear and move. In 2004, Gerstenfeld et al. reported a mouse DO model using an oral alveolar bone track distractor attached to the bone with 0.010-inch ligature wire fixation [2]. However, the ligature wire cannot guarantee the reproducibility of the model. In recent years, RISystem™ developed a commercially available device for the DO model, the MouseDis. MouseDis can be attached to the bone with 4 threaded pins. This device allows bidirectional distraction, which is not consistent with clinical situations. MouseDis fixator has to be applied by manual drilling, which causes variations. Therefore, a stable, reliable, easy-to-use and reproducible mouse DO model is still lacking.

In this study, we established a murine femoral model of DO using a custom-designed titanium mini-lengthener and fixed it to the femur through bi-cortical threaded pins. Imaging, histological assessments, and behavioral tests were performed to evaluate the effectiveness of this device. The results showed that the mini external lengthener enabled establishing a reliable and reproducible murine DO model.

## 2 Materials and methods

### 2.1 Animal information

All animal experiments were approved by the Animal Experimental Ethical Committee of the local university (Approval No. 22-213-NSF). Male C57BL/6J mice aged 10–12 weeks were purchased from Zhuhai BesTest Bio-Tech Co,.Ltd (Guangdong, China). Mice were housed at 22–25 °C with a cycle of 12-h light and 12-h dark and free access to a pelleted commercial diet and water.

### 2.2 Design of the mouse external distraction device

**Figure 1:**
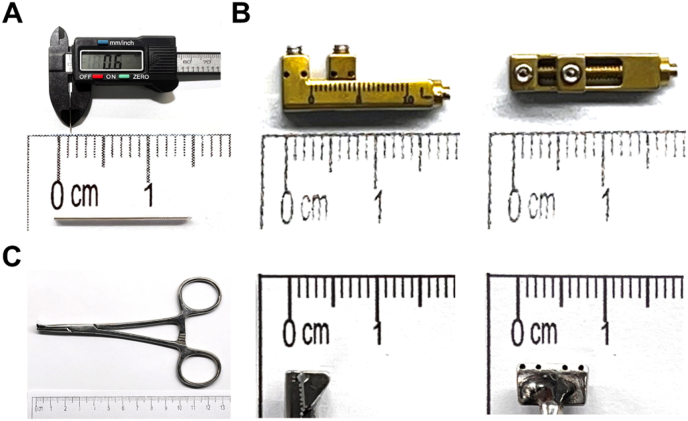
**Photographs of the surgical instruments and the mini-lengthener. A.** The diameter (top) and the length (bottom) of the threaded pins is 0.6 mm and 15 mm, respectively. The length of threaded portion of the threaded pin is 5 mm. **B.** Lateral view (left) and superior view (right) of the mini-lengthener. The length and the width of the mini-lengthener is 18.5 mm and 4 mm, respectively. **C.** Overview (left) of the guide clamp, lateral view (middle) and superior view (right) of the jaws of the guide clamp. The width of the extended jaws of the guide clamp 8.6 mm. The height of the closed extended jaws is about 5 mm.

The titanium mini-lengthener was customized and manufactured for the murine DO model (Fig. 1A). The mini-lengthener is composed of one L-shaped external fixator frame and four pins. The frame has one fixed end, which is used to lock two screws, and a movable unit for fixing the other bone segment after the osteotomy. The L-shaped frame has a sliding rod in the center that serves as an anchor for the sliding unit. A slotted head at the other end of the sliding rod is for manual adjustment of the sliding unit. Each clockwise turn of the slotted head will lead to a 0.4 mm movement of the sliding unit. The frame is fixed to the murine femur with 4 threaded pins (Fig. 1B). The threaded pin has a diameter of 0.6 mm and a length of 15 mm with a 5 mm threaded portion on one end. In addition, a customized drillguide clamp is used for easy position and drilling to ensure the consistent insertion of the threaded pins (Fig. 1C). This clamp consists of a surgical clamp and a drill guide with four vertical tunnels (0.8 mm in diameter and 5 mm in depth) for consistent position and drilling.

### 2.3 Surgical procedures

**Figure 2:**
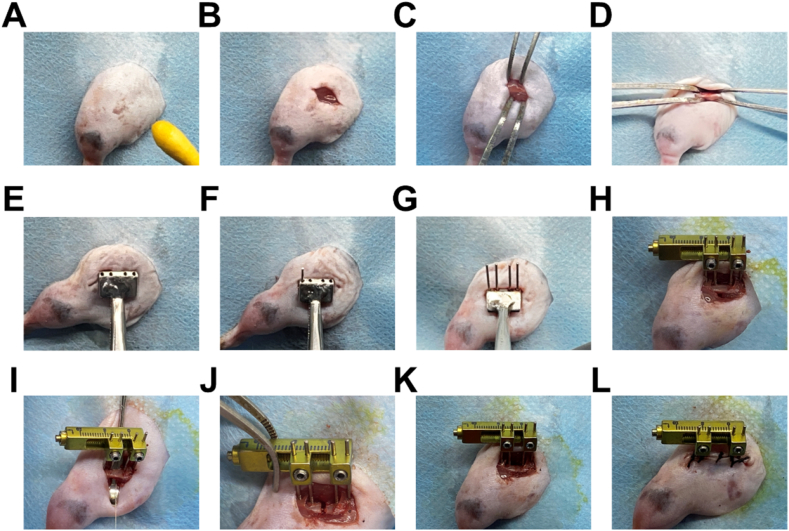
**Surgical procedure for installation of the mini-lengthener to the mouse femur. A.** Shave and sterilize the hind leg with iodophor. **B.** Incise the skin along the lateral intermuscular septum. **C.** Separate the biceps femoris and the vastus lateralis muscle by blunt separation. **D.** Expose the femur. **E.** Hold the femur with the guide clamp for drilling. **F.** After drilling, insert the first threaded pin at the distal lateral femur. **G.** Insert the second threaded pin at the proximal femur and the third and fourth pins at the diaphysis. **H**. Install the mini-lengthener after testing the stability of the pins. **I.** Secure the mini-lengthener. Place a dental photosensitive knife beneath the femur to protect the surrounding soft tissue during sawing. Gently pass the Gigli saw between the photosensitive knife and the femur. After the osteotomy, a tiny gap formed between the separated bone segments. The gap could be enlarged by clockwise turning the slotted head of the mini-lengthener (**J**). **K**. Close the gap between two bone segments. Irrigate with normal saline before wound closure. **L**. Close the fascia lata and the skin.

Animals were anaesthetized with 75 mg/kg ketamine (Alfasan International B.V., Holland) and 10 mg/kg xylazine (Alfasan International B.V., Holland). The animal was positioned in lateral recumbency. The left hind limb was shaved and sterilized with iodophor (Fig. 2A). After finger palpation to locate the lateral intermuscular septum between the biceps femoris and the vastus lateralis muscle, the skin was incised along the septum down to the fascia lata (Fig. 2B). The femur was exposed by incision on the fascia lata to separate the biceps femoris and the vastus lateralis muscle (Fig. 2C and D). Before applying the guide clamp, bluntly separated the biceps femoris and quadriceps femoris muscle from the femur [12]. The femur was held by the clamp (Fig. 2E). The distal side of the clamp was placed proximal to the lateral epicondyle. 4 holes were drilled through the tunnels on the drill guide using an electric drill with a 0.4 mm twist drill bit. Drilling should be performed vertically to the femoral surface to ensure that the pins are inserted parallelly with good alignment (Fig. 2F and G). Two hexagon socket head bolts were tightened to secure the threaded pins (Fig. 2H). Before osteotomy, the femur was isolated from the surrounding soft tissues. A transverse osteotomy was performed using a Gigli wire saw (0.1 mm diameter) (Fig. 2I and J) for precise cutting and creating flat cutting edge. The osteotomy gap and the wound were irrigated with saline (Fig. 2K), and the fascia lata and skin were closed sequentially with 5-0 suture (Fig. 2L) [13].

### 2.4 Femoral distraction protocol

**Figure 3:**
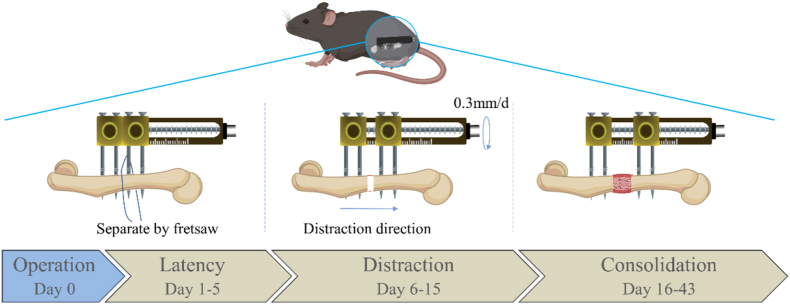
**Schematic illustration of the gradual distraction protocol.** The mini-lengthener was installed on the mouse femur. The gradual distraction protocol consisted of 5 days of latency after the osteotomy, followed by 10 days of distraction at a rate of 0.3 mm per day and 28 days of consolidation. Samples were collected at POD 43. POD, postoperative day.

The animals were randomly divided into 5 groups: the control group (Con), the sham group (Sham), the osteotomy with fixator-only group (FF), the acute lengthening group (AL), and the osteotomy with fixator and distraction (FFD) group. In the Con group, mice did not undergo any anesthesia or surgery. In the Sham group, the skin of mice was incised, the femur was exposed using blunt separation, the fascia lata was closed and the skin was sutured. In the FF group, mice with osteotomy were fixed with the mini-lengthener for 43 days. In the AL group, a 3-mm lengthening was performed immediately after the 5-day latency period, and the group waited for 38 days. In the FFD group, after 5 days of latency period, the gradual lengthening started at 0.3 mm (3/4 turn) per day for 10 days and waited for 28 days. All mice were terminated, and samples were collected 43 days post-osteotomy for further assessments (Fig. 3).

### 2.5 Digital radiography

Digital radiograph images of the operated femurs were obtained on postoperative day (POD) 5, 15, 22, 29, and 43. Images were acquired on a benchtop system consisting of an X-ray tube (L9421-02, Hamamatsu, Japan) and a flat-panel detector (Dexela 2923, Dexela, UK). Image acquisition was performed at 40 kV, 134 μA, with an exposure time of 2000 ms.

### 2.6 Micro-computed-tomography (μCT) scanning

Microstructural change within the distraction gap (regenerate) was qualitatively and quantitatively assessed using a μCT scanner (Skyscan 1276, Bruker, Kontich, Belgium). Briefly, the harvested femurs were fixed in 4 % PFA. After 24 h, the pins and external frame were removed carefully. The femurs were fixed in a custom-made holder and immersed in 70 % ethanol for scanning. Image acquisition was performed at 70 kV and 200 μA. Images were acquired at a resolution of 8 μm per voxel. The acquisition time was 350 ms. 2D images were reconstructed using NRecon software version v1.7.4.2 (Bruker, Kontich, Belgium). For quantitative analysis of the distraction gap, 121 continuous slices between the central two pins covering the distraction callus were selected as the volume of interest (VOI). Bone volume to total volume ratio (BV/TV) and bone mineral density (BMD) (g/cm^3^) were analyzed using CTan software v1.20.8 (Bruker, Kontich, Belgium). 3D images were reconstructed using CTvox software version v3.3.1 (Bruker, Kontich, Belgium).

### 2.7 Histological staining

The femurs were decalcified in 10 % buffered ethylenediaminetetraacetic acid for 2 weeks at room temperature. Then, the bones were dehydrated using gradient ethanol, vitrified using xylene, and embedded in paraffin. Bones were sectioned to 5 μm slides along the long axis. Hematoxylin and eosin (H&E) (Sigma–Aldrich, St Louis, MA), safranin-O/fast green (SO-FG) (Sigma–Aldrich, St Louis, MA) staining and Tartrate-resistant acid phosphatase (TRAP) staining (Sigma–Aldrich, St Louis, MA) with fast green counterstaining were performed according to standard protocols.

### 2.8 Immunohistochemistry staining

For immunohistochemistry staining, dewax and rehydrated slides were placed in antigen retrieval solution (pH 9.0) for 2 h at 65 °C. Sections were treated with 3 % hydrogen peroxide for 10 min to eliminate endogenous peroxidase activity. Sections were incubated with 0.3 % triton/phosphate buffered saline for penepetration. After blocking with 3 % bovine serum albumin (B265991, Aladdin, Shanghai, China) for 1 h at room temperature, sections were incubated with rabbit anti-osterix (OSX) (1:200, Ab209484, Abcam, MA, USA) overnight at 4 °C. After washing with phosphate buffered saline for 3 times, slides were incubated with horseradish peroxidase-conjugated goat anti-rabbit antibody (1:2000, Ab205718, Abcam, MA, USA) for 1 h at room temperature. After washing with phosphate-buffered saline 3 times, slides were visualized using a diaminobenzidine staining kit (ZLI-9017, ZSGB-BIO, Guangdong, China). Images were acquired using a widefield microscope (Olympus Slideview VS200 microscope, Japan).

### 2.9 Behavioral test

Mice were handled for 3 continuous days before the behavior test day. Motor activity was measured in an open field test box (50 cm in length × 50 cm in width × 40 cm in height) at POD 36. On the test day, mice were placed gently in the center of the plastic box and allowed to explore the box for 7 min. The activity of mice was recorded with a camera placed above the area. Video recording of the 2–7 min was analyzed, locomotion was quantified using the Anymaze™ software (Global Biotech Inc., USA). After each test, the apparatus or the arena was cleaned with 75 % ethanol and dried thoroughly before the next test.

The rotarod test was performed to assess motor function at POD 36. Prior to data collection, mice were trained on a rotarod apparatus at 4 rpm for 5 min each. After 3 times of training, the test started 1 h later. Mice were placed on the rotarod with gradually increasing speed, from 4 rpm to 40 rpm in 5 min. The latency to fall from the rod was recorded within this 5-min test period. The mean value of the 3 latencies was used for analysis. Mice received consecutive 3 trials with a 20 min rest between each trial. The rods were cleaned with 70 % ethanol after each trial.

### 2.10 Statistics

All statistical analyses were carried out using GraphPad Prism 9 software. Data were expressed as means ± SD and were analyzed using student t-test (lateralized rotational behavior) or one-way analysis of variance (ANOVA) with Tukey's posttest (μCT analysis, body weight, locomotion and rotarod test). *P* < 0.05 were considered statistically significant.

## 3 Results

### 3.1 A mini-external lengthening device was successfully manufactured for the mouse femoral DO model

A titanium mini-lengthener was customized for the mouse femur. With a length of 18.5 mm, a width of 4.0 mm. and a mean weight of 0.9551 ± 0.01119 g (Fig. 1). The mean weight of the 4 threaded pins was 0.03157 ± 0.000981 g. The total weight of the lengthening system was less than 1 g (5 % of the body weight of a 10-week-old male mouse, which was about 20 g). A customized guide clamp was designed. The guide clamp ensures vertical drilling and easy insertion of the threaded pins. The distance between the center of the outer two holes on the drill guide is 6.6 mm (Fig. 1), which is much shorter than the length of the mouse femur (mean lengths 14.50 ± 0.08 mm for male mice aged 12 weeks [14]). The application of the guide clamp minimized the area of the femur to be exposed for the surgery.

### 3.2 The imaging and histological examinations indicated a superior bone formation in the mouse DO model

**Figure 4:**
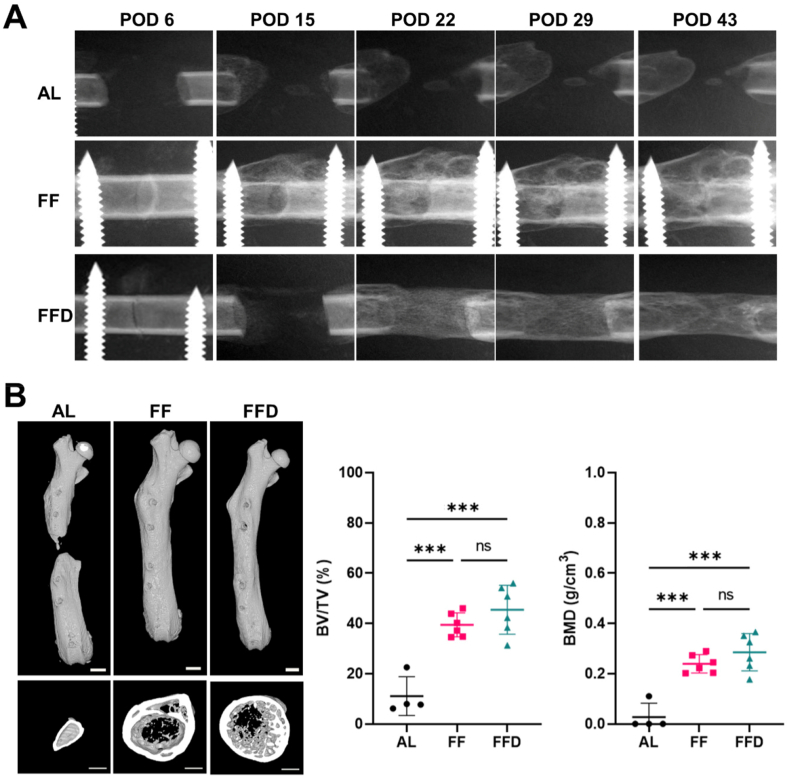
**Bone regeneration in the gap between separated bone ends in mice underwent femoral distraction osteogenesis. A.** Representative of digital radiographies of the operated femurs showed significantly more bone formation in the FF and FFD groups comparing to the AL group. Images were obtained at POD 6, 22, 29 and 43, respectively. **B.** Representative micro-CT images and quantitative analysis of operated femurs showed significantly higher BV/TV and BMD in the FF and FFD groups than those in the AL group. Samples were collected at POD 43 (n = 4 for AL group, n = 6 for FF and FFD groups). Scale bar, 500 μm; ns, no significance; ∗∗∗*P* < 0.001; error bars, SD; BV/TV, bone volume to total volume ratio; BMD, bone mineral density.

All mice recovered from the anesthesia after the surgery. All animals withstood the study. The right-sided femur was lengthened successfully. No mouse had infection or was lost. The mini-lengthener was stable and reliable in latency phase, distraction phase and consolidation phase. At POD 6, lengthening was completed for the AL group; the gap area was a radiolucent band, whereas the two bone ends were kept in contact in the FF group. Gradual distraction started for the FFD group, a narrow gap area could be observed between bone ends (Fig. 4A). At POD 15, there was a slight sign of periosteal new bone formation from the proximal bone end in the AL group; periosteal new bone formation was evident in the FF group; and lengthening was completed in the FFD group with a radiolucent band in the lengthening gap (Fig. 4A). At POD 22, 29 and 43, the bone gaps were largely radiolucent with some periosteal reaction adjacent to the bone ends in the AL group. In the FF group, the size of the periosteal reaction gradually enlarged, but the fracture gap was still visible, whereas in the FFD group, the radiolucent interzone was filled entirely with new bone at POD 22. The new bone was gradually consolidated with a homogeneous callus at POD 29, and the lengthening gap was completely united at POD 43 (Fig. 4A). In all groups, the external fixator/lengthener did not change position, the bone segments were aligned well and remained parallel to the intended lengthening line, and there was no sign of pin loosening (Fig. 4A).

At POD 43, Micro-CT images of the AL group showed an absence of bone regeneration in the lengthened gap, while the osteotomized bone united in the FF group and the lengthening gap was filled with remodeled and solid bone in the FFD group (Fig. 4B). Upon quantitative analysis, the BV/TV and BMD were significantly higher in the FFD group compared to those of the AL group (Fig. 4B).

**Figure 5:**
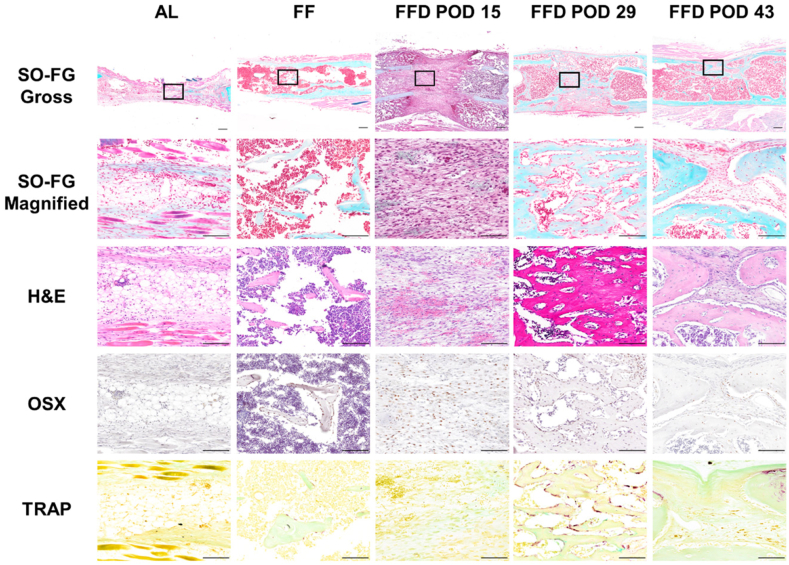
**Histological and immunohistochemistry analysis of the regenerates.** At POD 43, fat-like tissues but no sign of bone formation were shown in the AL group (column 1). Reestablished medullary canal and cortical bone were seen in the FF group (column 2). In the FFD group, after completion of lengthening (column 3), fibrous tissue was found in the distraction gap, with a great amount of OSX (+) osteoblasts but little TRAP-stained osteoclasts, indicating limited bone remodeling. FG-stained trabeculae filled the distraction gap at 2 (column 4) and 4 (column 5) weeks of consolidation. Osteoblasts and osteoclasts lined along the surface of newly formed trabeculae at POD 29 and the contacting surface of the separated cortical bones at POD 43, indicating active bone remodeling. The magnified portion is denoted by black frames. Scale bar, 300 μm.

At POD 43, the center of the distraction gap was full of fat-like tissues with no sign of bone formation in the AL group (column 1, Fig. 5). In the FF group, the osteotomy site was united with a reestablished medullary canal and cortical bone (column 2, Fig. 5). In the FFD group, H&E staining showed fibrous tissues within the interzone at the end of the distraction phase, and OSX-positive, spindle-shaped fibroblast-like cells filled the distraction gap, the cells were well-organized, aligned predominantly parallel to the distraction force within the distraction gap, a small amount of cartilage tissues were seen in the distraction gap, TRAP staining showed no sign of bone remodeling at the end of the distraction phase (POD 15, column 3, Fig. 5). At 2 weeks of consolidation (POD 29, column 4, Fig. 5), the fibrous interzone was replaced by well-organized-regenerated new bone trabeculae; a large amount of OSX (+) osteoblasts and TRAP-stained osteoclasts were seen along the surface of the newly formed bone, indicating active bone remodeling in the trabeculae. At 4 weeks of consolidation (POD 43, column 5, Fig. 5), the medullary canal was reestablished, the osteoblasts and osteoclasts were mainly seen at the contacting surfaces of the separated newly formed bones, indicating active bone remodeling in the remaining new bones. The histological feature of this mouse DO model replicates the well-established rat femoral DO model and other reported mouse DO models [15,10].

### 3.3 Measurement of potential complications in the mouse femoral DO model

**Figure 6:**
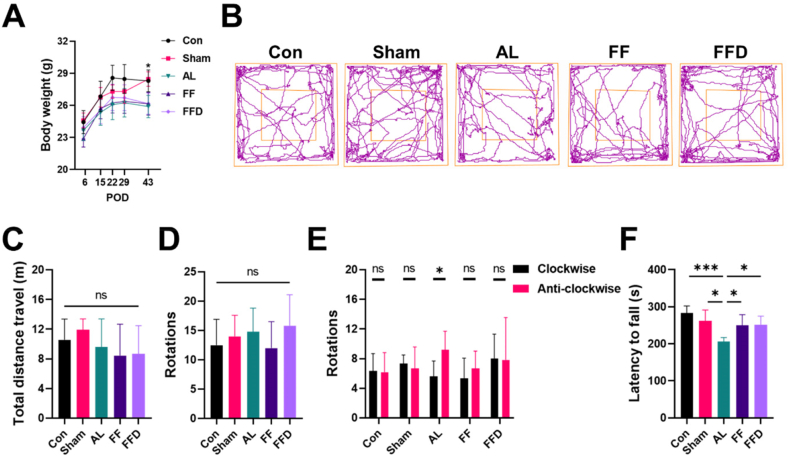
**Locomotion in the murine model of DO was not impaired. A.** The body weight of the Con, Sham, AL, FF and FFD groups measured at POD 5, 15, 22, 29 and 43. **B.** Representative trackplots and quantitative analysis showed no significant differences between groups in total distance travel (**C**) or total rotation number (**D**). More clockwise than anti-clockwise rotation was found in the AL group, which was not found in the Con, Sham, FF and FFD groups at POD 36 (**E**) (n = 3 for sham group, n = 5 for AL group, n = 6 for Con, FF and FFD groups). **F.** Quantitative analysis of latency to fall in the rotarod test showed significantly lower latency to fall in the AL group than in the Con, Sham, FF and FFD groups at POD 43 (n = 4 for sham group, n = 5 for Con, AL and FFD groups, n = 6 for FF group). ns, no significance; ∗*P* < 0.05, ∗∗∗*P* < 0.001. error bars, SD.

We monitored the potential complications associated with DO in the mouse model, including body weight and locomotor function. All mice tolerated the fixator well and could move following recovery from anesthesia. Mice that underwent osteotomy (the AL, FF and FFD group) had lower body weight after surgery compared to the Control (Con) and the Sham group. At POD 43, the body weight was significantly lower in all surgery groups (Fig. 6A). The body weight within the AL, FF and FFD groups showed no significant difference at all time points. These data suggested that more energy consumption was required for mice underwent surgery and bone repair.

Locomotion is a critical factor in bone regeneration, and potential side effects on the locomotion of mice were evaluated using the open-field tests (OFT) and the rotarod tests. Results from OFT showed no significant differences in total travel distance and the number of total rotations between each group (Fig. 6B–D). Considering that the surgery was performed on the unilateral femur, the number of clockwise and anti-clockwise rotations were analyzed. While mice in the Con, Sham, FF and FFD groups did not show lateralized rotational behavior, mice in the AL group preferred anti-clockwise rotations compared to clockwise rotations, indicating an imbalance between operated and unoperated legs (Fig. 6E). We next assessed the locomotor ability using the rotarod test [16]. Mice in the AL group showed reduced latency to fall from the rotarod, indicating impaired locomotor ability. Mice in the Con, Sham, FF and FFD groups did not show a significant difference in latency to fall between groups (Fig. 6F). Above results suggested that the Con, Sham, FF, and FFD groups did not suffer from reduced locomotion ability, whereas the AL group showed imbalanced locomotion and impaired locomotor function.

## 4 Discussion

Previously, the DO animal models were mostly established in dogs, rabbits or rats but rarely in mice. In this study, we successfully developed a reproducible murine femoral DO model. Using the customized external fixator and the guide clamp, the alignment between bone segments were well-maintained during the lengthening and consolidation period, and the DO treatment did not cause significant impairment in the locomotor function of the animals.

There were two types of mouse DO models in the literature: the use of circular external fixators and the use of unilateral external fixators [17]. The first mouse DO model was reported [9,10], in which the mouse tibia was fixed with two circular rings, with pins for transfixing the bone and attached to the frame. Using a circular frame in mice was difficult, and the surgery was time-consuming. There was a long learning curve to establish this mouse DO model reliably. The other mouse DO model employed a straight-type external fixator, such as an oral alveolar bone distractor [2]. We have attempted to secure an oral alveolar bone distractor to the mouse femur using threaded pins and dental cement. However, free-hand drilling caused variability in pin alignment, and the lengthening outcome was not consistent (Supplementary Fig. 1); hence, we abandoned using this animal model. Recently, the MouseDis system has been commercially developed by the RISystem™*,* which is a rigid unilateral external fixator/lengthener secured to the femur by bi-cortical pins, allowing controlled lengthening and stability [13]. Similarly, our mouse DO system employs 4 bi-cortical pins and two hexagon socket head bolts for fixation, which enhance stability and rigidity. In contrast to the RISystem™ mouse lengthener, which allows movement of both the proximal and the distal bone segments, our system only moves the distal bone segment, which simulates clinical scenarios and provides a more stable mechanical environment for bone formation.

In addition, our costumed guide clamp allows the efficiency of the surgery and reproducibility of the mouse DO model, which has not been proposed in any of the previously reported mouse DO models. During the surgery, the femur is held steady using the guide clamp for drilling and insertion of the threaded pins. The four vertical tunnels in the drilling guide are parallel to each other, allowing easy vertical drilling and quick insertion of the threaded pins. This will ensure the perfect alignment of the bone ends during the lengthening phase. What's more, the fixed distance between each tunnel guarantees the position of the four pins inserted into the femur to enable more rigid fixation. The use of the customed surgical guide clamp allows the consistent location of the pins and osteotomy site, which are essential for the reproducibility of the model.

We have also evaluated the effects of our device and DO procedure on the mobility of the mice because locomotion is a critical confounding factor in bone regeneration and DO [3,18]. The animal behavioral tests showed that our device and the DO procedure did not impair animals' well-being and mobility during the DO treatment. In contrast to bulkier circular frames that might interfere with weight bearing and animals’ mobility, our device is light, with a total weight of about 1 g, and it is user-friendly; animals can bear weight on the operated limb and move freely 24 h after surgery.

The clamp with a drilling guide makes the mouse DO model relatively easier and reproducible, minimizing operator variations. However, attention should be paid to avoid injuries to the sciatic nerve and the femoral artery. When applying the clamp, muscles around the femur shall be carefully stripped, and there should be no soft tissues between the bone and the clamp's contact tips. The drilling shall be done at a low speed and stopped immediately after penetrating the second cortex to avoid damaging the underlying soft tissues.

For bone quality assessments, micro-CT and histological examination results showed well-organized newly formed bone trabeculae in the FFD group at POD 15, 29 and 43, while no bone formation but adipocytes-like tissues were seen in the distraction gap in the AL group, suggesting that acute lengthening impaired bone regeneration and should be avoided.

In summary, we have developed a unilateral mini-lengthener with a drilling guide for establishing a mouse femoral DO model. The mouse DO model is reliable and reproducible and duplicates the biological processes of DO with no impairment to the animal's well-being and locomotor ability. This mouse DO model may be used for in-depth study of the molecular mechanisms of DO using various transgenic mice.

## Authorship contribution statement

YJ. L, JM. Y and ZW. J designed the study, performed major experiments and data analysis. M. W, HX. W, XT. Z, X. L, SS. B, J. S, L. Z, DS. G and BT. L provided administrative, technical or material support, and interpreted the results. YJ. L, JM. Y and SE. L drafted the manuscript. SE. L, F. Y and G. L supervised, conceived the study and edited the manuscript. All authors read and approved the final manuscript.

## Declaration of competing interest

All the authors declare no conflicts of interest with the contents of this article.

## Abbreviations

DO: Distraction osteogenesis
FF: Fracture Fixation
AL: Acue lengthening
FFD: Fracture with fixator and distraction
POD: Post-operative day
μCT: Micro-Computed-Tomography
VOI: Volume of interest
BV/TV: Bone volume to total volume ratio
BMD: bone mineral density
H&E: Hematoxylin and eosin
SO-FG: Safranin-O/fast green
TRAP: Tartrate-resistant acid phosphatase
OSX: Osterix
Con: Control
OFT: Open-field tests
