# Supplementary material for: Development of a standardized and reproducible murine femoral distraction osteogenesis model
Source: J Orthop Translat. 2024 Oct 4;49:74–81. doi: 10.1016/j.jot.2024.08.001 (PMC11488447; doi:10.1016/j.jot.2024.08.001)
Supplement: Multimedia component 1 [file mmc1.docx]

**Original article**

*Supplementary material for*

**Development of a standardized and reproducible murine femoral distraction osteogenesis model**


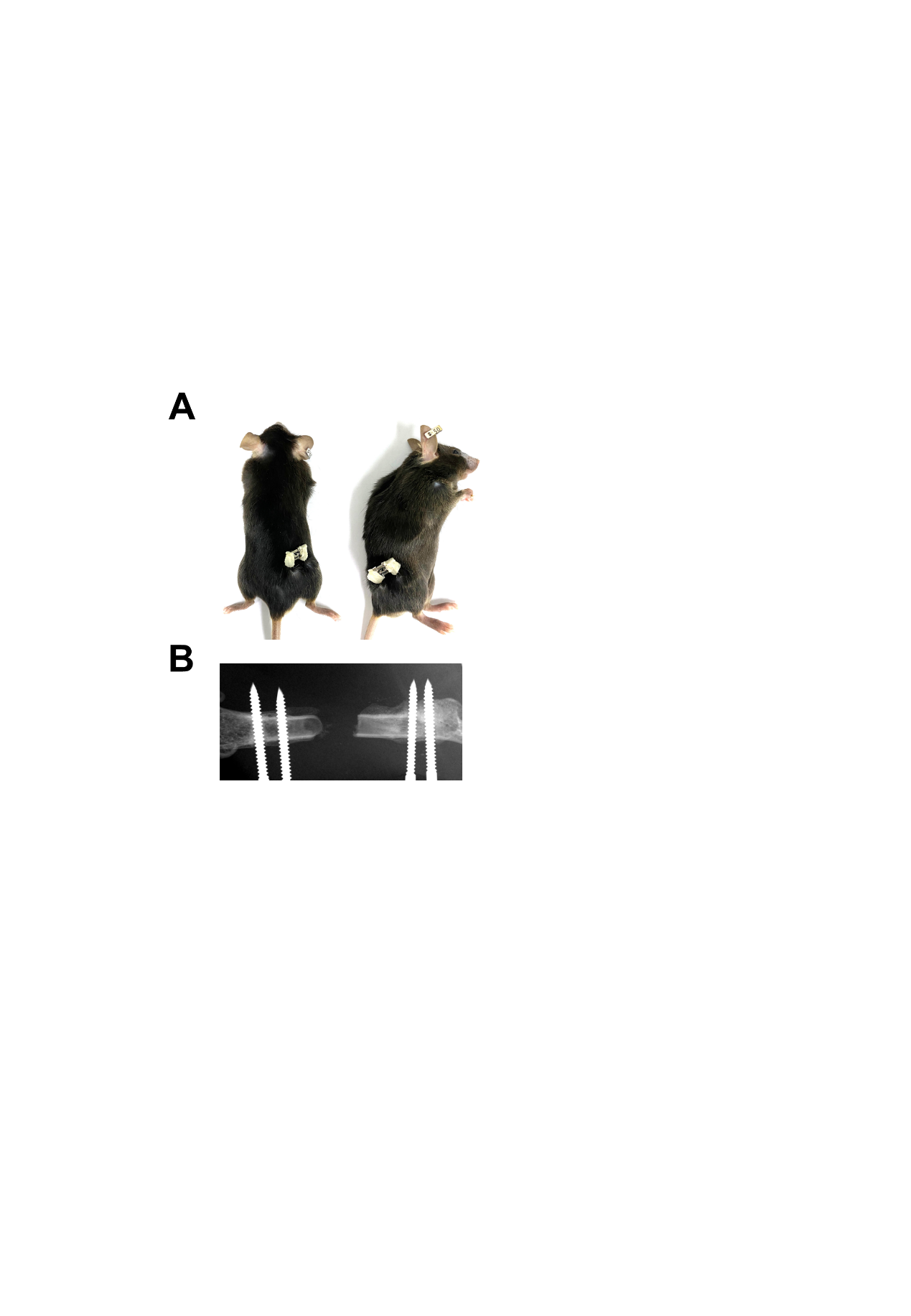


**Supplementary Figure 1. Murine femoral model of DO with an oral alveolar bone distractor as the external distraction device.** Representative photograph (Latency phase) (**A**) and digital radiography image (Distraction phase) (**B**) of mice installed with an oral alveolar bone distractor.
